# Framing, moral foundations and health taxes: interpretive analysis of Ethiopia’s tobacco excise tax policy passage

**DOI:** 10.1136/bmjgh-2023-012058

**Published:** 2023-10-09

**Authors:** Daniel Erku, Nigusse Yigzaw, Henok Getachew Tegegn, Coral E Gartner, Paul A Scuffham, Yordanos Tegene Garedew, Ehetemariam Shambel

**Affiliations:** 1 Centre for Applied Health Economics, Griffith University, Southport, Queensland, Australia; 2 Menzies Health Research Queensland, Griffith University, Gold Coast, Queensland, Australia; 3 Institute of Public Health, University of Gondar, Gondar, Ethiopia; 4 School of Rural Medicine, University of New England, Armidale, New South Wales, Australia; 5 School of Public Health, University of Queensland, Herston, Queensland, Australia; 6 Health Policy and Systems Research, EPIC Research and Training Institute, Addis Ababa, Ethiopia; 7 Pharmaceutical and Medical Equipment Directorate, Federal Ministry of Health, Addis Ababa, Ethiopia

**Keywords:** public health, health policies and all other topics, qualitative study

## Abstract

**Background:**

In 2019–2020, the Ethiopian government ratified a suite of legislative measures that includes levying a tax on tobacco products. This study aims to examine stakeholders’ involvement, position, power and perception regarding the Ethiopian Food and Drug Authority (EFDA) bill (Proclamation No.1112/2019). This includes their meaning-making and interaction with each other during the bill’s formulation, adoption and implementation stages.

**Methods:**

We employed a mixed-methods design drawing on three sources of data: (1) policy documents and media articles from government and/or civil society groups (n=27), (2) audio and video transcripts of parliamentary debates and (3) qualitative stakeholder interviews.

**Results:**

Policy actors in both the public health camp and tobacco industry employed several framing moves, engaged in distinctive patterns of moral rhetoric, and strategically invoked moral languages to galvanise support for their policy objectives. Central to this framing debate are issues of public health and the danger of tobacco, and the protection of ‘the economy and personal freedom’. The public health camp’s arguments and persuasiveness—which led to the passage of the EFDA bill—centred around discrediting tobacco industry’s cost–benefit assessments through frame disconnection, or by polarising their own position that the financial, psychological and lost productivity costs incurred by tobacco use outweighs any tax revenue.

**Conclusions:**

A successful cultivation of an epistemic community and engagement of policy entrepreneurs—both from government agencies and civil society organisations—was critical in creating a united front and a compelling affirmative policy narrative, thereby influence excise tax policy outcomes.

**WHAT IS ALREADY KNOWN ON THIS TOPIC**

- The Ethiopian government ratified one of Africa’s strongest anti-tobacco laws (Proclamation No.1112/2019), which includes levying an excise tax on tobacco products.
- Understanding stakeholder’s involvement, position, power and perception regarding health taxes, and their meaning-making and interaction with each other is central to health policy.
- However, little is known about how the policy agenda was set, who was involved in the agenda setting, and what impact the political environment had in setting, shaping and/or influencing Proclamation No.1112/2019.

**WHAT THIS STUDY ADDS**

- This article provides insights on how stakeholders have grappled with and responded to policy controversies over the issue of tobacco excise tax policy in Ethiopia, and how policy actors move beyond reductionist frames of ‘health’ and ‘economy’.
- Engaging civil society organisations early on—based on a shared understanding, role and responsibilities—is critical in formulating a compelling affirmative policy narrative, thereby influence excise tax policy outcomes.
- Understanding the wider political economy landscape and identifying or capitalising on favourable policy conditions is crucial for fast-tracking excise tax policy passage.

**HOW THIS STUDY MIGHT AFFECT RESEARCH, PRACTICE OR POLICY**

- Our findings enhance our understanding of how framing dynamics shape health tax policy adoption and demonstrates opportunities of using moral foundational framing interactions to understand how different perspectives are formulated, (re)framed, communicated and understood by various actors.

## Background

Tobacco, alcohol and sugary beverage consumption accounts for more than 10 million premature deaths each year globally (about 16% of deaths).^1^ People from low-income and middle-income countries (LMICs) bear a disproportionate share of the associated health and economic consequences.^2 3^ Evidence from around the world demonstrates that implementing well-designed excise tax policies will reduce the consumption of tobacco and alcohol and saves lives.^2 4–6^ In addition, pro-health taxes can substantially boost government revenue and reduce future healthcare costs by curbing the growth of the non-communicable diseases that these products can cause. This is of critical importance in the post-COVID-19 era, as many sub-Saharan African countries including Ethiopia are facing serious economic contraction.^7^ Generating additional tax revenue while reducing health risks will reduce strain on public finances and boost available funding for essential health services.^2 8^ However, health taxes are underused policy tools in many LMICs because affected industries vigorously oppose tax increases with false or misleading claims related to their impact on revenue, employment and illicit trade.^2 9^

In 2019, the Ethiopian government announced and ratified a suite of legislative measures that restricts the use, sale and advertisement of alcohol and tobacco. In February 2020, the Ethiopian parliament passed a new bill introduced by the Finance Minister to standardise taxation (Excise Tax Proclamation No. 1186/2020).^10^ The bill introduced, among other things, a tax system for tobacco products generally in line with WHO recommendations.^11^ The Excise Tax bill was broader in scope than only tobacco (ranges from automobiles and watches to goods and services that are believed to be luxury, hazardous to health).^12^ This legislation comes after Ethiopia’s parliament unanimously approved one of Africa’s strongest antitobacco laws in 2019 (Ethiopian Food and Drug Authority (EFDA) Proclamation No.1112/2019, also known as EFDA bill),^13^ which requires the Ministry of Finance to levy a tax on tobacco products consistent with the WHO Framework Convention on Tobacco Control (WHO FCTC). Given the broader scope of the bill (ie, includes sections related to food, medicine, alcohol and tobacco products), the legislative process attracted unprecedented attention from the public, media, public and private sectors. In what was characterised as a ‘historical’ bill, the parliament held, for the first time, a series of public hearings and broadcasted parliamentary debates on a national television. The bill mandates, among other things, an excise tax on tobacco products consistent with the WHO FCTC. While the Ministry of Finance is responsible for implementing excise tax proclamation No. 1186/2020, the EFDA, which operates independently under the Ministry of Health, carries out the ratification of the WHO FCTC, to which Ethiopia has committed, hence the name EFDA bill. Although the policy-making processes advancing this legislation were shaped by complex cross-border interorganisational networks including the WHO and several civil society groups, little is known about how the policy agenda was set, who was involved in the agenda setting, and what impact the political environment had in setting, shaping and/or influencing the policy agenda. Understanding the political economy of health taxes can help governments to best position and frame health taxes politically and overcome potential barriers accelerating implementation.^14–16^ The aim of this study is to examine stakeholder’s involvement, position, power and perception in passage of this legislation (ie, Proclamation No.1112/2019 and Excise Tax Proclamation No. 1186/2020). This includes actors’ interaction during the formulation and adoption stages. While the legislation had a broader scope and content (ie, includes sections related to food, medicine and tobacco products), we focus our analysis on tobacco products.

## Methods

We employed a mixed-methods design and relied on three main sources of data: (1) policy documents from government and/or civil society groups and media articles; (2); audio and video transcripts of parliamentary debates and (3) key stakeholder interviews. Written informed consent from participants was also obtained before conducting this study. Participants’ identifying information obtained was kept anonymous.

### Policy and media documents

Fiscal legislative documents, public submissions to parliamentary inquiries and government discussion papers related to the draft Food and Drug Authority Proclamation No.1112/2019 (hereafter called EFDA Bill) and the Excise Tax Proclamation No. 1186/2020 were retrieved from official government websites (eg, the Ministry of Health, the EFDA and Ministry of Finance) and civil society groups (eg, the Campaign for Tobacco-Free Kids). These data were supplemented with a manual search of the five most popular newspapers in Ethiopia based on website traffic and readership ranking (ie, The Reporter, Addis Standard, Addis Fortune, BBC Amharic and The Africa Report) for articles and media stories regarding fiscal legislation, covering the time before, during and after legislative passage. Only articles that were published between September 2014 and January 2022, and those that addressed factors or actor involvement relating to the passage of the tobacco excise tax were included. A total of 27 documents (11 policy documents and 16 media articles) were included based on inclusion criteria and their relevance to the study aim.

### Secondary data from parliamentary debate and public hearings

After conducting the first reading in 2018 on the proposed EFDA bill (Bill No. 13/2011, later became Proclamation No.1112/2019), the House of People’s Representatives referred the bill to two Standing Committees: (1) Standing Committee for Trade and Industry and (2) Standing Committee for Women, Youth and Social Affairs for detailed review and examination. Subsequently, the joint Standing Committee: (1) organised a forum and invited relevant officials (eg, Ministry of Health, EFDA) to answer questions from members of the House, (2) invited various stakeholders through written letters and national broadcasting media to participate in the bill, in which 27 stakeholders submitted their position statements and (3) organised two public hearings in which members of the public raised questions and comments and appropriate responses and explanations were given by officials from the Ministry of Health and the EFDA. The debate from public opinion forums and public hearings were recorded and broadcast on local broadcasting media. In this study, we analysed stakeholder’s written submissions to and the transcripts of public hearings from the parliamentary debate and public hearings.

### Key informant interviews

Key informants for semistructured in-depth interviews and focus group discussion were identified through review of policy documents (eg, legislative documents, minutes of proceedings, proposals, presentations and reports), snowballing, personal networks of the research team and input from local partners and policy-makers via stakeholder engagement meetings. This approach allowed us to identify visible stakeholders (eg, individuals, legislators, bureaucrats, politicians, academics, civil society and non-governmental organisations) who participated in the policymaking process and/or possessed extensive knowledge of pro-health taxes in Ethiopia. Key informants were invited via email, written letter and phone calls. An open-ended interview guide was prepared based on the concepts of the selected theoretical framework. Participants were asked to reflect on the processes leading up to the legislative passage, including what happened to their own and others’ framing moves in and around decisive moments in the policy passage process. A total of 17 stakeholders were interviewed by DE, ES and HGT between May 2022 and July 2022 in Addis Ababa, Ethiopia (ie, in-depth interview with five key informants and three focus group discussions, each consisting of four key informants). Interviews lasted on average between 30 min and 1 hour, whereas the focus group discussion lasted up to 2 hours. All interviews were conducted in Amharic and audio recorded using a portable digital recorder. Interviews were conducted over the phone (n=3) and face to face in a convenient place comfortable to interviewees (n=2). Recordings from interviews and focus group discussions were transcribed and translated using a professional transcription and translation service.

Stakeholder analysis was conducted to comprehensively examine the roles, interests, positions and interactions of key actors involved in the legislative process of the EFDA bill. The first step involved the identification of relevant stakeholders through an extensive review of existing literature, policy documents and expert consultations. Next, a thorough role analysis was conducted to understand the formal positions, responsibilities and functions of each stakeholder within the policy process. This analysis also considered their informal roles, including their influence, expertise and relationships with other stakeholders. Position analysis and interaction mapping exercise was performed to examine the specific stance, priorities and potential conflicts or alignments of each stakeholder and identify patterns of collaboration, conflict or cooperation among stakeholders. This analysis involved reviewing their public statements, policy proposals and actions related to the EFDA bill, providing insights into their position within the policy landscape.

### Theoretical framework and analytical approach

Health taxes can be understood (and represented) in different ways. Within the public health community, health taxes may be understood as a population health measure to reduce mortality and morbidity. Governments may also see health taxes as a means to generate revenue and/or to expand fiscal space. For the industries affected by health taxes, health taxes represent a negative impact on their businesses. It is useful to understand the approaches different actors use to shape interpretation of policies and examine the differences in sense-making between people, actors, or organisations. This process—known in the policy analysis arena as issue framing—is instrumental in learning how actors make sense of decision problems, change processes and their conflictive interactions with other actors.^17–19^

Framing is a complex and contested phenomenon with profound implications.^20 21^ It serves as an interactive mechanism for selecting or omitting prominent aspects of our social reality, thereby facilitating shared comprehensions of policy issues and their respective solutions.^21 22^ Frames can be differentiated based on their function: whether they define, diagnose, assess or prescribe solutions.^23^ In the domain of policy analysis, framing is principally embedded in the postpositivist literature, which employs interpretive and critical methods to examine policy-making as a contested meaning-making activity.^24^ This scholarship links framing—in its form and function—closely to metaphorical concepts,^25^ causal narratives^26^ and policy issues.^27^ These perspectives underscore the crucial role language and symbolic expressions play in policy processes.^28 29^ Informed by this approach, interpretive policy analysts seek to understand the conditions and processes under which specific frames emerge and persist.^30^ Dewulf and Bouwen^31^ suggest an interactional approach to framing, emphasising the construction of meaning through discourse. They view framing as ‘the dynamic enactment and alignment of meaning in ongoing interactions’ and frames as ‘transient communication structures built around issues during conversational turns’.^31^ They propose five interactional framing mechanisms: (1) incorporation, (2) accommodation, (3) disconnection, (4) polarisation and (5) reconnection.^31^Frame incorporation involves integrating a softened reformulation of a challenging element into one’s own issue framing. Frame accommodation involves strategically adjusting one’s viewpoint to align with the challenging facets of an issue. This could entail revising, redefining, or even downgrading one’s original framing to aptly engage with intricate elements within the issue. Frame disconnection entails detaching the challenging element from the ongoing discourse, labelling it as irrelevant or unimportant. Frame polarisation emphasises the disparity by reaffirming one’s own issue framing or an upgraded version of it. Frame reconnection involves acknowledging both elements seriously and mitigating the incompatibility between them. Research indicates that these argument and framing developments (discursive, cognitive or otherwise) often stem from and are shaped by individuals’ moral foundations and values.^32–34^ Framing, in its function, summons potent emotional responses by incorporating deeply entrenched morals and societal values into the rationale for political action.^35^

Social psychologists propose that framing is a primary means through which moral judgements shape intuition, consequently leading to social persuasion.^36^ Despite its significant role, the moral underpinnings of these judgments often remain underrepresented in much of the health policy framing discourse, including the degree to which frames underscore universally significant themes such as fairness vs cheating or loyalty versus betrayal.^17 31^ Not all moral values and the frames based on them carry equal significance. The level of importance assigned to each moral foundation can vary considerably among individuals. This partially explains why some people are more perceptive to a certain frame than others. However, arguments can become much more effective if (re)framed in a way that aligns with and appeals to the target audience’s most important moral values. In this study, we propose that framing strategies, rooted in specific moral foundations, empower policy entrepreneurs—individuals or groups actively involved in shaping and influencing policy outcomes^37^—to effectively challenge, reformulate, reinforce or resolve policy controversies. Drawing on interactional framing mechanisms^31^ and moral foundation theory^33^ and using an interpretive policy analysis approach,^38^ we evaluated: (1) the frame-convergent and frame-divergent framing moves adopted by actors and policy entrepreneurs in the lead up to the passage of the above two bills, (2) the underling moral foundations invoked and the moral language used in a policy debate and (3) the impact of these framing contests on shaping the final policy outcome. All included documents and interviews were coded using NVivo V.12 software. We developed an initial codebook based on our research questions and theoretical frameworks. Two authors piloted the codebook and conducted initial analyses with three sample (two policy documents and one interview). Coding discrepancies were discussed and addressed before one researcher (DE) coded the remaining interviews and documents. Legislative documents on each bill’s history and status were used to develop the tobacco excise tax policy timeline.

### Patient and public involvement

Our study did not involve enrolment of patients or members of the public.

## Results

### Sense-making and the role of policy entrepreneurs

As an agency created by the House of Peoples’ Representatives to implement the WHO FCTC, the EFDA led the drafting, advocacy and implementation of the EFDA bill under a experienced senior policy team composed of lawyers and public health experts led by Ms Heran Gerba (Director General of EFDA) and Dr Amir Aman (Minister, Minister of Health). As the initiator of the EFDA bill (and a key partner in the excise tax bill), EFDA were an adept actor, building a strong camp and cultivating an international or national epistemic community with the other actors. The design and legislative process of the EFDA bill, tabled by the EFDA and approved by all members of the parliament, took place over a period of 2 years from 2018 to 2019 (table 1).

**Table 1:** Timeline and overview of tobacco control legislations (including tax policies) in Ethiopia

Year	Proclamation(s)	Policies and main contents
Pre 2014	Proclamation 30/1942	Tobacco Regie Proclamation was enacted in 1942 to monopolise the preparation, manufacture, importation, distribution and export of tobacco products
	Proclamation 197/1980, Proclamation 181/1999	The National Tobacco and Matches Corporation was established and mandated to exclusively grow and process tobacco in Ethiopia, later transferred to the National Tobacco Enterprise share Company.
	Proclamation 661/2009, Regulation 299/2013 Proclamation 759/2012	Food, Medicine and Healthcare Authority of Ethiopia (now Ethiopian Food and Drug Authority (EFDA)) was authorised to regulate tobacco products, and in 2012, EFDA prohibited advertisement and promotion of cigarette or other tobacco products.
	Excise Tax Proclamation No. 307/2002	The government’s excise tax proclamation taxed ‘luxury and basic goods which are demand inelastic’ based on the production cost of products (as opposed to ex-factory price).
2014–2018	Proclamation 822/2014	Ethiopia ratified the WHO FCTC, and Food, Medicine and authorised the Healthcare Authority of Ethiopia to take all necessary steps for its implementation.
	NTCD 28/2015	The Food, Medicine and Healthcare Authority of Ethiopia issued the National Tobacco Control Directive No 28/2015 (NTCD) in accordance with Article 4 of the FCTC. The first draft of the National Tobacco Control Strategic Plan was prepared in 2017 under the direction of the authority.
2018 – present	EFDA Proclamation No.1112/2019	Ethiopia’s parliament unanimously approved the EFDA bill, which requires, among other things, levying a tax on tobacco products consistent with the WHO FCTC recommendation.
	Excise Tax Proclamation No. 1186/2020	The Federal Parliament approved a new Excise Tax repealing Excise Tax Proclamation No. 307/2002 and its amendments. (‘Repealed Proclamation’). Drafted by the Ministry of Finance, the New Proclamation introduces new concepts and excise tax rates including a 30% tax rate of the cost of producing cigarettes, in addition to a specific excise rate of 8 Ethiopian Birr on each individual packet.

WHO FCTC, WHO Framework Convention on Tobacco Control.

### Framing, moral foundations and discursive strategies

Through a comprehensive stakeholder analysis, taking into account roles, interests, positions and interactions within the policy context, we identified two prominent camps that were prominent during the legislative process: the public health camp spearheaded by EFDA and the tobacco industry camp consisting of commercial actors, trade associations and affiliated entities. The public health camp’s arguments and persuasiveness—which led to the passage of the EFDA bill—was visible through contextualised and carefully formulated interactional framing mechanisms, supported by evidence. When dealing with differences with agencies outside of the camp (eg, parliamentarians, ministry of finance), policy entrepreneurs within the EFDA-led camp tended to formulate their framing around accommodation, reconnection or incorporation. Below, we highlight some of the framing mechanisms employed by both camps.

### Dealing with tobacco industry’s dystopian narrative: disconnection and polarisation

The tobacco industry’s opposition to the proposed policies created a policy dystopian narrative highlighting, reaffirming and exaggerating the ‘unintended, yet unavoidable’ costs of the policy (in terms of the economy, law enforcement and social justice), while the potential benefits of the proposed policies are underplayed and discredited. Both the public health camp and tobacco industry used the strategy of frame polarisation to strengthen their position statements. Framing contests and arguments between the two camps took place primarily in the parliamentary debate, but also in the mainstream media domain, where they amplified their policy claims and polarised the issue. Emphasising the ‘role of the tobacco industry in creating jobs and paying taxes’, tobacco industry representatives (and affiliates) warned of negative economic impacts of the proposed excise tax policy claiming that the excise tax bill will force them to out of the market and create unemployment. A representative from National Tobacco Enterprise said ‘*…In fact, [our] tobacco company pays more than 1.2 billion Birr to the government, perhaps the health care budget will probably come from here… there is not a single article in this bill that controls [illicit traders] who smuggle illicit tobacco and import guns and rifles with the money’.*

The impact of the proposed policy on reducing tobacco industry’s revenue was not directly presented as a concern in and of itself (ie, deemphasised), but their arguments were rather framed—often by third parties—as a genuine concern to the country’s economy and employment rate, mediated through reduction in sales, jobs and tax payments. Similarly, the policy was opposed on the grounds that it was disproportionate and inconsistent with internationally accepted legislation. Referring to the WHO FCTC, a representative from the Addis Ababa Chamber of Commerce and Sectoral Associations argued that the bill is inconsistent with international tobacco control laws (in addition to domestic or international trade and investment laws), accusing proponents of ‘becoming more Catholic than the pope’. Another key and frequent argument was the potential impact of increasing illicit trade. Employing a frame disconnection strategy, the tobacco industry questioned the credibility of the evidence, invoking this both as a rhetorical concept and by referring to their own evidence. The current empirical evidence was described by a representative from National Tobacco Enterprise as ‘*inconsistent and too small to draw credible inference on the effectiveness of the proposed policy solutions in curbing smoking rates’.* Proportionality arguments were also connected to denials of or discrediting the evidence base underlying the policy. Referring to a study conducted by National Tobacco Enterprise on illicit trade which claimed that 44% of the cigarettes smoked in Ethiopia are smuggled cigarettes, the company’s representative said: ‘*Had this report reached the minister beforehand, it is my belief that the honourable minister would have revised the proclamation that was presented today’.*

The above discursive strategies and (re)framings were accompanied with a suite of moral values and foundations. The tobacco industry, as an opponent of the proposed bills, relied almost exclusively on the endorsement of liberty (ie, personal freedom) and authority foundations in their moral arguments against passing the bills. Throughout their discursive languages, smoking was portrayed as a matter of individual choice and liberty, manifested, for example, in an emphasis on consumer choice and a rhetorical shifting of responsibility away from the companies that produce and market tobacco products. Introducing the proposed bill was portrayed as stripping people of their liberty and a threat to civil rights and an indication of social injustice. A representative from Addis Ababa Chamber of Commerce and Sectoral Associations argued: ‘*we must not forget that this is a matter of personal freedom, especially for the person over 18 years of age, and apart from informing the public about the risk of product use, the government cannot and should not act as a guardian by saying what to do and what not to do’.*

However, the above framing moves and discursive strategies employed by the tobacco industry were not successful. Policy entrepreneurs within the public health camp either outright rejected tobacco industry’s claims and cost–benefit assessments through frame disconnection, accompanied with evidence or by polarising their own claim—again backed by scientific evidence and lived personal experiences—that the financial, psychological, and lost productivity costs incurred by tobacco use outweighs any tax revenue. By carefully wording discursive languages, (re)framing arguments and strategically appealing to the public’s and parliamentarian’s moral intuitions, the public health camp managed to generate support for their framing. The public health camp first highlighted the urgent need for protecting the lives of ‘*more than 30 million children’* [representative from Mathewos Wondu Ye Cancer Society] from the tobacco toll (ie, maximising care and minimising harm for children) and imposing excise tax for ‘*the greater good’* (representative from Mathewos Wondu Ye Cancer Society). In one of the key strategic moves, the public health camp engaged a former National Tobacco Enterprise Director turned avid advocator for tobacco control. In the parliamentary debate and written submission, Mr. Wondu, who left the tobacco industry and established a cancer civil society organisation after losing his son to cancer, described his personal experience with tobacco, and tied it with the need to protect the next generation of children. In a televised parliamentary debate, Mr. Wondu said: ‘*I have worked at the Monopoly [National Tobacco Enterprise] for 14 years and smoked for 17 years. I know, firsthand, the impact of tobacco. This country has more 30 million young people, and the parliament should do everything in their power to pass this legislation, save a generation and make this country a good place to live and work.’*

This was followed by rejecting the tobacco industry’s argument of ‘consumer choice’, by questioning the moral authority of the industry which was described by the public health camp as ‘*the only industry that manufactures a product which* kills *up to half of its consumers’* [representative from Mekuamia]. In some instances (eg, during the parliamentary debates), both camps engaged in a ‘‘tit for tat’’ strategy whereby the tobacco industry invoked ‘liberty’ and ‘authority’ moral values and the public health camp, responded by either rejecting or reformulating the moral rhetoric in their own terms and to their own advantage. A representative from Mathewos Wondu Ye Cancer Society argued: ‘*The tobacco industry is talking about personal freedom, mind you, what we are saying is that 3.4 million Ethiopians are smoking tobacco, and no one is talking about them’.*

As part of the frame polarisation strategy, the public health camp invited two representatives from the Addis Ababa Children’s Parliament to comment on the impact of the bill on their future as the next generation of Ethiopians. The EFDA-led camp also invited renowned artists and actors, some of whom formerly smoked very heavily, to describe their own experiences. Mr. Fantu Mandoye, a famous Ethiopian actor, who previously drank alcohol and smoked heavily, described for members of the parliament the reason for quitting smoking as the following: ‘*I went to a store with 15 cents to buy Niyala cigarette, but they said ‘no, it has increased to 20 cents’ And with that, I stopped smoking and have been smoke-free for the last 31 years.’*

These rhetorical strategies were effective in influencing parliamentarians to the extent that some members of parliament questioned the rationale behind having cigarettes on the market in the first place, some suggesting ‘*sun-setting and tobacco endgame’* policies should be considered (a member of the parliament in a televised parliamentary debate). The overwhelming acceptance of the bill by the public and members of the parliament was also partially attributed to the fact that tobacco smoking is considered a taboo or socially undesirable practice, and as such, it was relatively easy for the public health camp to appeal to parliamentarian’s moral intuition.

### Standing on common ground: increasing excise tax rate through reconnection

When the excise bill was first proposed in 2014, the specific additional rate on each packet of cigarettes was just 5 Ethiopian Birr (ETB), calculated based on modelling study conducted by WHO and the World Bank in 2014–2015 (unpublished documents). However, adjusted for inflation, the rate is considered far below WHO’s recommended rate in year 2019. The public health camp advocated for adopting a higher excise tax rate taking into consideration inflation rate and the recommended 10% yearly tax increment recommended by WHO. Initially, state minister of the Ministry of Finance referred to the high illicit trade figure that was produced by the National Tobacco Enterprise (NTE) as the main reason not to impose the required amount of excise tax, essentially invoking some of the most common discursive strategies used by the tobacco industry. In a series of technical workshops on tobacco tax modelling and open and closed meetings with high-level policy-makers, the public health camp (with WHO as the main partner) carefully studied the Ministry of Finance’s rationale underpinning their stances and reframed the issue along the lines of the additional revenue that could be generated, and healthcare costs saved by increasing the excise tax from 5 ETB to at least 8 ETB. The EFDA-led camp treated expanding fiscal space as an issue element in its own right, for which the proposed increase excise tax rate will bring relief. Rather than directly dealing with original excise tax rate difference, the issue was dealt with by reconnecting the fundamental priority for the government (ie, enhancing revenue generation) to the original story, indirectly through the intermediary element of increasing the excise tax rate. Here, several key policy entrepreneurs within the camp (mainly from civil societies) were instrumental in formulating and reformulating these framings and in successfully negotiating a workable solution (by means of frame reconnection) while simultaneously taking EFDA’s and the Ministry of Finance’s issue framing seriously. As part of the reconnection process, key policy entrepreneurs proactively reached out to tje Ministry of Finance office and key members of the parliament’s Standing Committee and provided evidence that although illicit trade is prevalent in the country, it has, in fact, more to do with border control than with increasing excise tax (thus discrediting the illicit trade argument). This sustained advocacy effort and issue reframing during the weeks that followed the initial adoption of the excise tax draft led to the final legislation reflecting WHO recommendations more fully (ie, 30% plus 8 ETB per pack of cigarettes).

### Reframing ‘earmarking’ through accommodation

An interesting turn of framing happened by way of accommodation. Once the EFDA-led camp achieved its objective of passing the EFDA bill, they continued to advocate for earmarking any tax revenue collected from tobacco excise tax to finance public health priorities, including for providing smoking cessation services. However, given the government’s many competing priorities, it was difficult for the public health camp to convince the Office of Prime Minister and other stakeholders to earmark the excise tax. But most importantly, the earmarking argument was challenged by the government on the grounds that such earmarking practices are new to Ethiopia and for earmarking to be practical, the government would need to repeal several other laws. When asked about earmarking arguments, the Chair of Standing Committee for Women, Youth and Social Affairs said: ‘*The issue of earmarking may not go as they intended, but the camp have the potential to initiate a change in the underling legislative bottlenecks and make this a reality—it just takes time, but for now, they should celebrate this big win’.*

Cognizant of the legal challenges and impracticalities of earmarking, the public health camp accommodated their framing with that of the government by quickly reformulating their discursive languages around ‘an equitable distribution of the pooled excise tax funds’ and to give due consideration to ‘the immense physical, psychological, social and economic toll that tobacco causes’ when distributing these funds. This way, the public health camp managed to make their issue framing acceptable for the other, while maintaining the coherence in their own framing (ie, without taking back some of their earlier statements about earmarking).

## Discussion

Understanding stakeholder involvement, position, power and perception regarding the proposed policy, and their meaning-making and interaction with each other is central to health policy.^39^ We examined stakeholder’s meaning-making and interaction—through frame contestations—during the formulation and adoption stages of the EFDA bill and excise tax legislation. Central to this framing debate are issues of public health and the danger of tobacco, and the protection of ‘the economy and personal freedom’. Both camps employed a number of framing moves, engaged in distinctive patterns of moral rhetoric (although placing different weight on the foundations) and strategically invoked moral languages to galvanise support for their policy objectives. Ethiopia’s experience with the passage of EFDA bill and excise tax bill offers several insights and learning for other LMICs. First and foremost, understanding the broader political and economic landscape and identifying or capitalising on favourable policy conditions is vital for the expedited passage of excise tax policies. Since Ethiopia ratified the WHO FCTC in 2014, the issue of health tax—particularly for tobacco products—has been on top of the ministry of health’s agenda.^40^ Many organisations including the WHO and World Bank generated a suite of evidence highlighting the need for policy change and provided capacity building to local government agencies and policymakers.^41^ However, between 2014 and 2018, there was no significant change in tobacco control and excise tax policy. In 2018, a favourable climate for policy change emerged with the election of a new government in Ethiopia, which promptly embarked on an ambitious initiative to transform the political environment by launching significant economic reforms. In September 2019, this new administration unveiled its ‘Homegrown Economic Reform Plan’, serving as a concrete roadmap to initiate macro, structural and sectoral reforms.^42^ At the forefront of this reform agenda was a strategy to enhance domestic revenue mobilisation via tax policy and administration reforms. This significant shift in policy direction facilitated the passage of one of Africa’s most stringent anti-tobacco laws (the EFDA Bill), and expedited the ratification of the Excise Tax Proclamation No. 1186/2020. Both of these measures mandate an excise tax on tobacco products by the relevant government body.

The tobacco industry’s distorted cost–benefit arguments were rather met by Parliamentarians with either fierce criticism or refusal to engage with the ‘unfounded’ claims. This was possible due to the public health camp’s strong, sustained and evidence-based counter claim—framed as polarisation and/or disconnection—that the excise tax bill will, in fact, result in substantial benefit for the economy, the government and society at large. Early on in 2014, the public health camp prepared a comprehensive ‘bottom-up’ advocacy plan that empowered and gave ‘voice to the voice less’ such as children and the general public. Engaging civil society organisation early on—based on a shared understanding, role and responsibilities—was critical in presenting a strong, united front. EFDA’s successful cultivation of an epistemic community and engagement of several policy entrepreneurs and policy champions—both from government agencies and civil society organisation—is one of the secrets behind its compelling affirmative policy narrative, and is an example of how alliances, argumentation, and policy levers can be streamlined to influence policy outcomes. A similar strategy was employed by public health camp in Argentina, where networking with strategic partners and policy champions and presenting a united front via an agreed on ‘communications strategy’ were effective in adopting an excise tax on tobacco products, leading to a 50% increase in average retail price of.^43 44^

The legislative outcome for EFDA bill was not only shaped by framing and argumentation, but also by a variety of instrumental, action-based strategies. From countering a skewed evidence base underpinning projected policy failure to directly interfering with the implementation of the bill, the tobacco industry attempted to block, weaken and/or delay the ratification of the bills.^45^ For example, despite the EFDA bill forbidding a government entity from accepting any assistance from the tobacco industry on any enforcement activities or entering into any partnership with it, the Custom Commission, under the Ministry of Revenue, signed a Memorandum of Understanding with the National Tobacco Enterprise (part-owned by Japan Tobacco International) to fight illicit trade in June 2019.^45 46^ In addition, despite the Ministry of Council granting the Ministry of Finance to impose 10% annual tax increasement on ‘health damaging products’, there is no excise tax change over tobacco products in 2021. While the reason is unclear, it is likely that the industry is using the illicit trade argument to persuade the government and weaken tobacco taxation. Tobacco industry interference with tobacco control—from design and adoption to implementation of excise tax and other policies—has been widely reported in the literature.^47–51^

### Strength and limitations

Our study contributes to the literature on frame contestation by empirically analysing the frames on the issue of excise tax policy. By unpacking how different meanings and perspectives are formulated, (re)framed, communicated and understood by various actors, our analytical approach offers unique insights into sense-making in tobacco excise tax policy, how actors restructured policy conversation through a choice of framing strategies. In the context of health taxes, studying how various stakeholders act informed by their action frames and respond to the frames of others—often underpinned by moral values—allows us to examine not only the origins and evolution of the frames but also how policy actors move beyond reductionist frames of ‘health’ and ‘economy’. While the findings of our study contribute valuable insights to the understanding of policy framing in the context of Ethiopia’s tobacco excise tax policy, there are certain limitations that need to be acknowledged. In our analysis, we employed moral foundations theory (MFT) to help identify and understand the moral arguments that underpin some of the framing strategies used in the context of Ethiopia’s tobacco excise tax policy. This focus on moral arguments is premised on the notion that moral considerations often appeal to deeply ingrained values and beliefs, thereby having a profound influence on individuals’ attitudes towards and actions concerning a policy. However, we acknowledge a potential limitation of this approach. By focusing on MFT, we potentially risk underemphasising other aspects of industry framing strategies which may not be readily explained or accounted for within the purview of MFT. Moreover, we understand that the effectiveness of framing strategies grounded in MFT may depend on a comprehensive understanding of the audience and its diversity. The appeal of specific moral principles can vary within a population, making the framing strategies less universally effective. Hence, in this study, our intention is not to tailor arguments to specific policy actors but to illuminate the role of moral arguments in shaping broader policy discourses. Moreover, we have integrated what might appear as conflicting frameworks: MFT and interpretative policy analysis (IPA). The inherent universal principles advocated by MFT may appear in stark contrast to the antifoundational position of IPA, which accentuates the conditional and constructed nature of social phenomena, including morality. Nonetheless, we posit that these theories, despite their visible dichotomy, can be harmoniously and complementary employed. Our paper is an effort in this direction. We apply MFT to discern overarching moral themes related to health taxes and use IPA to grasp how these themes are uniquely interpreted and enacted within Ethiopia’s tobacco excise tax policy landscape. Despite the inherent theoretical discordance, our work affirms that these methodologies can work in synergy, thereby contributing to a more profound and nuanced analysis.

The exploration of the tobacco industry’s influence on policymaking is a complex endeavour, fraught with methodological and ethical challenges. The tobacco industry is characterised by a high degree of opacity, making it challenging to procure and verify data. Further, the industry’s framing strategies are often subtle and nuanced, requiring careful and rigorous analysis. Our dataset holds a significant limitation; the majority of our in-depth interviewees were aligned with the public health perspective, due to difficulties recruiting industry respondents. Despite these hurdles, we’ve endeavoured to maintain the accuracy and comprehensiveness of our data and analysis, incorporating the industry’s viewpoints drawn from inquiries and media coverage on tobacco products. These constraints inevitably bound our study’s breadth and depth, a fact we acknowledge, and we hope that future research will further explore these critical areas. Our study was also limited to analysing the passage of the EFDA and excise tax bills, with a particular focus on tobacco products, and did not capture recent attempts by the industry to reduce the annual tax increase.

## Conclusions

This article presents an interpretive policy analysis of how policy actors and stakeholders have grappled with and responded to policy controversies over the issue of tobacco excise tax policy in Ethiopia. A successful cultivation of an epistemic community and engagement of policy entrepreneurs—both from government agencies and civil society organisations—is critical to present a strong, united front and a compelling affirmative policy narrative, thereby influence excise tax policy outcomes. Using tobacco excise tax as an example, our findings enhance our understanding of how framing dynamics shape health tax policy adoption. There is a need for more research to better understand how moral foundational framing interactions can be used to understand how various stakeholders act, informed by their action frames and respond to the frames of others, and how policy actors move beyond reductionist frames of ‘health’ and ‘economy’.

## Data Availability

All data relevant to the study are included in the article or uploaded as online supplemental information.
